# Selective inhibition of liver X receptor α-mediated lipogenesis in primary hepatocytes by licochalcone A

**DOI:** 10.1186/s13020-015-0037-x

**Published:** 2015-04-21

**Authors:** Gyun-Sik Oh, Gang Gu Lee, Jin Yoon, Won Keun Oh, Seung-Whan Kim

**Affiliations:** Department of Pharmacology, Asan Medical Center, University of Ulsan College of Medicine, Seoul, 138-736 Korea; Bio-medical Institute of Technology, University of Ulsan College of Medicine, Seoul, 138-736 Korea; Korea Bioactive Natural Material Bank, College of Pharmacy, Seoul National University, Seoul, 151-742 Korea

## Abstract

**Background:**

Sterol regulatory element binding protein-1c (SREBP-1c) is a regulator of the lipogenic pathway and is transcriptionally activated by liver X receptor α (LXRα). This study aims to investigate phytochemicals inhibiting the autonomous transactivity of LXRα with potentials as SREBP-1c inhibitors. Licochalcone A (LicA) is a flavonoid isolated from licorice root of Glycyrrhiza plant.

**Methods:**

The effects of 238 natural chemicals on autonomous transactivity of LXRα were determined by the Gal4-TK-luciferase reporter system. The inclusion criteria for chemical selection was significant (*P* < 0.05) inhibition of autonomous transactivity of LXRα from three independent experiments. Transcript levels of mouse primary hepatocytes were measured by conventional or quantitative RT-PCR. Luciferase assay was used to assess synthetic or natural promoter activities of LXRα target genes. The effect of LicA on lipogenic activity was evaluated by measuring cellular triglycerides in mouse primary hepatocytes. The recruitment of RNA polymerase II to the LXR response element (LXRE) region was examined by chromatin immunoprecipitation.

**Results:**

Among 238 natural compounds, LicA considerably inhibited the autonomous transactivity of LXRα and decreased the LXRα-dependent expression of SREBP-1c. LicA inhibited not only LXRα-dependent activation of the synthetic LXRE promoter but also that of the natural SREBP-1c promoter. As a consequence, LicA reduced the LXRα agonist-stimulated transcription of several lipogenic genes. Furthermore, LXRα-dependent hepatic lipid accumulation was repressed by LicA in mouse primary hepatocytes. Interestingly, the LXRα-dependent activation of ATP-binding cassette transporter A1 (ABCA1) and ATP-binding cassette transporter G1 (ABCG1), other LXR target genes involved in reverse cholesterol transport (RCT), was not inhibited by LicA. LicA hindered the recruitment of RNA polymerase II to the LXRE of the SREBP-1c gene, but not of the ABCA1 gene.

**Conclusions:**

LicA is a selective inhibitor of LXRα, repressing lipogenic LXRα target genes but not RCT-related LXRα target genes.

## Background

Enzymes involved in lipogenesis, such as acetyl CoA carboxylase (ACC), fatty acid synthase (FAS), stearoyl-CoA desaturase 1 (SCD1), long-chain elongase, and glycerol-3-phosphate acyltransferase (GPAT) [1-7], can be rapidly controlled by posttranslational and/or allosteric mechanisms, but are mostly regulated on a long-term basis at the transcriptional level and particularly by sterol regulatory element binding protein-1c (SREBP-1c) [8,9]. SREBP-1c is a transcription factor of the basic-helix-loop-helix-leucine zipper family and is a regulator of the lipogenic pathway [10,11]. Transcription of the abovementioned lipogenic enzymes is augmented by SREBP-1c activation [8,10].

SREBP-1c appears to be the main regulator of lipogenesis in the liver but not in adipose tissue, as shown by a reduction in lipogenic enzymes in the liver but not in adipose tissue following SREBP-1c knockout [12]. In contrast to lipogenesis in adipose tissue, increased hepatic lipogenesis has many harmful effects, leading to nonalcoholic fatty liver disease and steatohepatitis [8,13]. Additionally, hepatic lipogenesis strongly correlated with insulin resistance [14,15]. Therefore, modulation of hepatic SREBP-1c activity could be exploited to treat such metabolic diseases.

SREBP-1c is transcriptionally activated by liver X receptor α (LXRα), which is a transcription factor belonging to the nuclear receptor family [16]. LXRα is also an important regulator of lipogenesis, as it directly regulates other lipogenic genes such as ACC, FAS, and SCD1, as well as SREBP-1c, and is associated with hepatic steatosis [17-19]. Thus, LXRα inhibitors could inhibit more lipogenic genes than SREBP-1c inhibitors.

In addition to lipogenic genes, LXRα regulates reverse cholesterol transport (RCT)-related genes including ATP-binding cassette transporter A1 (ABCA1) and ATP-binding cassette transporter G1 (ABCG1). In terms of metabolic disease, lipogenesis is considered harmful for fatty liver diseases, whereas RCT is considered beneficial for atherosclerosis. More specifically, while LXR agonists possess potent antiatherosclerotic effects, they cannot be used for antiatherosclerotic therapy because of lipogenic adverse effects. Thus, the selective regulation of LXR target genes is crucial as a therapeutic approach.

This study aims to investigate phytochemicals with inhibitory activity against the autonomous transactivity of LXRα for identifying SREBP-1c inhibitors. The selected LXRα inhibitors will be further elucidated for its underlying molecular mechanism.

## Methods

### Phytochemicals and reagents

Two hundred and thirty-eight natural plant chemicals were provided by Dr. Won Keun Oh (Korea Bioactive Natural Material Bank, Seoul National University, Seoul, South Korea). T0901317 and dimethyl sulfoxide (DMSO) were purchased from Sigma-Aldrich (St. Louis, MO, USA). Percoll was purchased from GE Healthcare (Pittsburgh, PA, USA). M199 medium was obtained from Gibco (Grand Island, NY, USA). Dulbecco’s Modified Eagle’s Medium (DMEM) and fetal bovine serum (FBS) were products of Hyclone (Logan, UT, USA).

### Preparation of mouse primary hepatocytes and cell culture

Mouse primary hepatocytes were prepared from 8–10-week-old male C57BL/6 mice (Orient Bio, Seongnam, South Korea) using a previously described collagenase perfusion procedure with minor modifications [20]. Briefly, the liver was perfused with Mg^2+^/Ca^2+^-free Hank’s balanced salt solution (Sigma-Aldrich) containing 100 U/mL collagenase type II (Gibco) and 48 μg/mL trypsin inhibitor (Sigma-Aldrich). Next, viable hepatocytes were harvested using Percoll density gradient centrifugation after filtering through a 100-μm nylon mesh (BD Biosciences, San Jose, CA, USA). Isolated primary hepatocytes were plated onto collagen type I-coated culture dishes and maintained in M199 media. Human hepatoma HepG2 cells were maintained in DMEM containing 10% FBS at 37°C in a humidified 5% CO_2_ incubator. All animal protocols were approved by the Institutional Animal Care and Use Committee (IACUC) of the Asan Institute for Life Sciences, Asan Medical Center (Approval No. 2013-12-168). Mice were maintained in a temperature-controlled facility with a 12 h light/12 h dark cycle and provided *ad libitum* access to water and regular rodent chow diet. Following acclimatization for 1 week, the mice were sacrificed for the preparation of primary hepatocytes.

### Transfection and luciferase reporter assay

HepG2 cells were plated into 24-well plates 24 h prior to transfection and grown in DMEM supplemented with 10% charcoal-stripped FBS. Transfections were performed with Gal4-TK-luciferase, Gal4-hLXRα ligand binding domain (LBD), 3 × LXRE-luciferase, SREBP-1c LXRE-luciferase, ABCA1 LXRE-luciferase, or ABCG1 LXRE-luciferase using Lipofectamine 2000 reagent (Invitrogen, Grand Island, NY, USA) according to the manufacturer’s protocol. After 24 h, the cells were treated with 1 μM of the LXR agonist T0901317 and 10 μg/mL of natural compounds for 18 h. Luciferase activity was then measured using a Centro LB 960 luminometer (Berthold Technology, Bad Wildbad, Germany), and enzyme activity values were normalized to β-galactosidase levels. For normalization, pActin-βgal plasmids were cotransfected into hepatocytes along with the promoter-luciferase reporter genes. The inclusion criteria for chemical selection was significant (*P* < 0.05) inhibition of autonomous transactivity of LXRα from three independent experiments.

### Analysis of transcript levels by conventional or quantitative RT-PCR

Total RNA was extracted from primary hepatocytes by Trizol-RNA Lysis Reagent (Invitrogen) according to the manufacturer’s protocol. Purified RNA was reverse transcribed using M-MLV reverse transcriptase (Promega, Madison, WI, USA). Next, specific mRNA levels were analyzed by conventional or quantitative RT-PCR. The oligonucleotide primers used for conventional RT-PCR were as follows: SREBP-1c, 5′-GGC GCA TGG ATT GCA CAT TT-3′ and 5′-GCA GGC TGT AGG ATG GTG A-3′; ABCA1, 5′-ACA AAG AGC TGG AGC GAC AT-3′ and 5′-CAG CCA ACT CCT GAA AGG TC-3′; and 18S rRNA, 5′-CGT CCC CCA ACT TCT TAG AG-3′ and 5′-CAC CTA CGG AAA CCT TGT TAC-3′. 18S rRNA was used as an internal control in conventional RT-PCR.

Quantitative gene expression analysis was performed on a LightCycler 480 system (Roche, Basel, Switzerland). The following oligonucleotide primers were used for quantitative RT-PCR: SREBP-1c, 5′-CAG CCA TGG ATT GCA CAT TTG-3′ and 5′-GTC TTG GTT GTT GAT GAG CTG G-3′; FAS, 5′-CTG CAG CTG TCA GTG TGA AGA AG-3′ and 5′-GCA GCA TTT TTA CCA GGT TGG T-3′; SCD1, 5′-AGC TCA GTC TCA CTC CTT CCC TTA-3′ and 5′-CAG CCA GCC TCT TGA CTA TTC C-3′; GPAT, 5′-TCA TCC AGT ATG GCA TTC TCA CA-3′ and 5′-GCA AGG CCA GGA CTG ACA TC-3′; ABCA1, 5′-GAC CCG TAC TCT CGC AGG G-3′ and 5′-CCT TGC CGG TAT TTT AGC AGG-3′; ABCG1, 5′-TTC ATC GTC CTG GGC ATC TT-3′ and 5′-GAA ATA GGC GAT GAG CCG C-3′; and ribosomal protein S29, 5′-CGC AAA TAC GGG CTG AAC A-3′ and 5′-GCC TAT GTC CTT CGC GTA CTG-3′. Ribosomal protein S29 was used as an internal control for quantitative RT-PCR.

### Cellular triglyceride (TG) accumulation

Primary hepatocytes were plated onto 60-mm collagen-coated culture dishes, incubated overnight in M199 medium, and treated with 1 μM T0901317 and 10 μg/mL LicA for 24 h. After treatment, the cells were washed twice with cold phosphate buffered saline (PBS), lysed in PBS containing 1% Triton X-100 for 30 min at 4°C, and centrifuged (Micro 17TR, Hanil Science Industrial, South Korea) at 10,000 × *g* for 10 min at 4°C. Cellular lipids were then extracted from the supernatant by the Bligh and Dyer method [21]. TG levels were determined by a TG assay kit (Asan Pharmaceutical, Gyeonggi-do, South Korea) and normalized to total protein levels.

### Chromatin immunoprecipitation (ChIP) assay

ChIP assays were performed on primary hepatocytes treated with 1 μM T0901317 and 10 μg/mL selected compound for 30 min, as described previously, with minor modifications [22]. Briefly, cells were fixed with 1% formalin (Sigma-Aldrich) for 20 min and then quenched with 0.125 M glycine for 5 min at room temperature. The cells were washed twice with cold PBS and lysed in SDS lysis buffer containing 50 mM Tris-HCl (pH 8.0), 10 mM EDTA, and 1% SDS. Soluble chromatin was prepared by sonication (VCX-600 sonicator, Sonics & Materials, Newton, CT, USA) and then pre-cleared by protein G-agarose beads (Santa Cruz Biotechnology, Santa Cruz, CA, USA). Pre-cleared supernatants were then immunoprecipitated with anti-RNA polymerase II (Santa Cruz Biotechnology) or IgG antibodies (Abcam, Cambridge, UK) for 12 h at 4°C. The final DNA extracted from the immunoprecipitate was analyzed by quantitative RT-PCR using the LightCycler 480 system (Roche). The oligonucleotide primers used for the ChIP assay were as follows: SREBP-1c LXRE region, 5′-AGG CTC TTT TCG GGG ATG G-3′ and 5′-TGG GGT TAC TGG CGG TCA C-3′; and ABCA1 LXRE region, 5′-GGG GAA AGA GGG AGA GAA CAG-3′ and 5′-GAA TTA CTG GTT TTT GCC GC-3′. Immunoprecipitated DNA levels were presented as fold enrichments normalized to 10% input DNA levels.

### Statistical analysis

Quantitative values were presented as means (SD). Statistical analysis was performed using one-way ANOVA (SPSS19, IBM, Chicago, IL, USA). For post-hoc analysis, Fisher’s LSD (least significant difference) test was conducted. Bonferroni method was used to correct *P* values for multiple comparisons. Differences with *P* values less than 0.05 were considered statistically significant.

## Results

### LicA inhibits the autonomous transcriptional activity of LXRα and LXRα-stimulated expression of SREBP-1c

**Figure 1:**
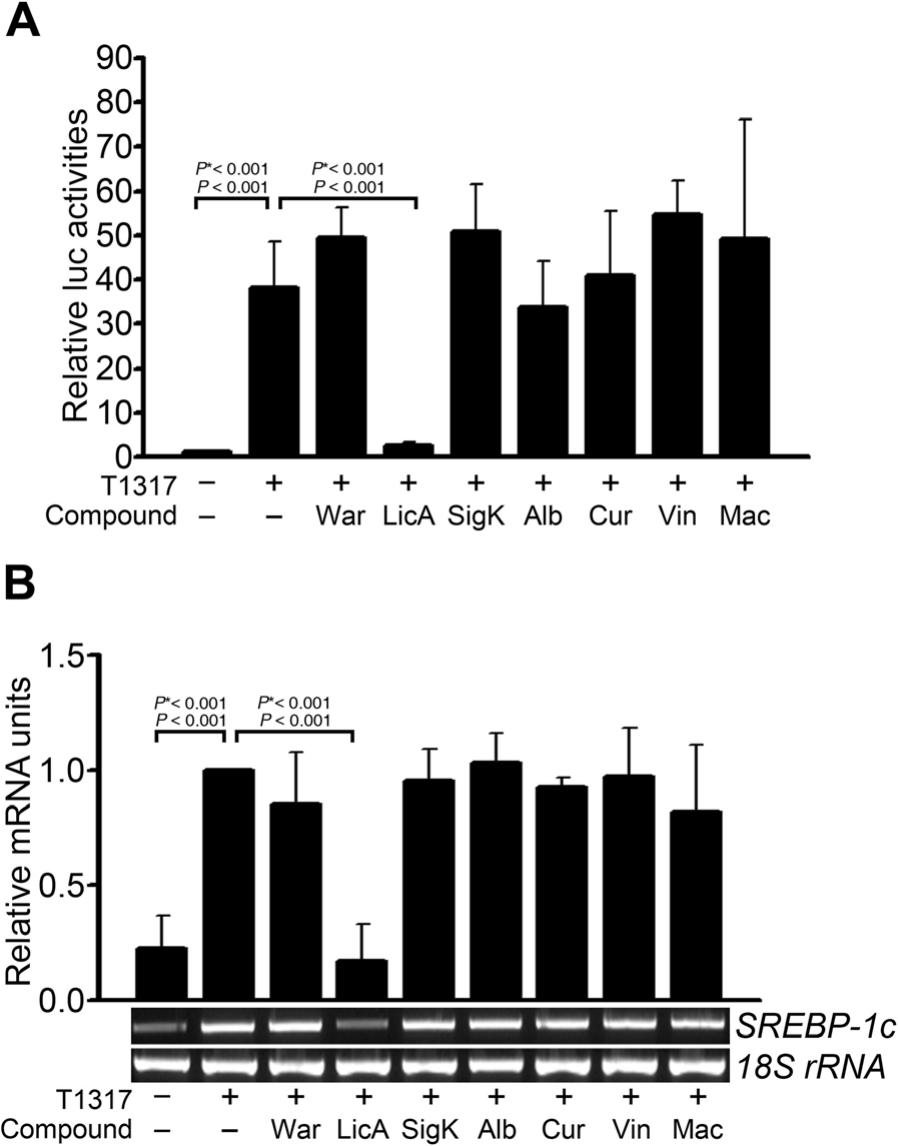
Effects of phytochemicals on **(A)** the autonomous Gal4-hLXRα LBD transactivity and **(B)** the LXRα agonist-stimulated transcript levels of SREBP-1c. **(A)** Gal4-LXRα LBD transactivity was measured in the presence of 1 μM T0901317 and 10 μg/mL natural compounds by a Gal4-TK- luciferase assay. Data were presented as means (SD) from three independent experiments with duplicate determinations. **(B)** Effects of natural compounds on T0901317-stimulated SREBP-1c mRNA levels in primary hepatocytes. mRNA levels were measured by conventional RT-PCR. Quantitation of band intensities was performed using ImageJ (NIH, Bethesda, MD, USA). Data are presented as means (SD) from three independent experiments. Statistical analysis was performed using one-way ANOVA. P* = *P* value for Bonferroni correction. T1317, T0901317; War, warangalone 4′-methyl ether; LicA, licochalcone A; SigK, sigmoidin K; Alb, albaspidin P-P; Cur, curcumin; Vin, ɛ-viniferin; Mac, macelignan.

Two hundred and thirty-eight compounds isolated from various plants were tested in a Gal4-dependent transactivation assay by Gal4-hLXRα LBD. Licochalcone A (LicA) showed strong inhibitory activity against the autonomous transactivity of LXRα (Figure 1A). Because SREBP-1c is a regulator of lipogenesis and an LXRα target, we then investigated the effects of these plant-derived compounds on the transcript levels of SREBP-1c to determine the antilipogenic effect of LicA. LicA also repressed LXRα agonist T0901317-stimulated transcription of SREBP-1c (Figure 1B).

### LicA directly modulates the promoter activity of synthetic LXRE and SREBP-1c LXRE reporters

**Figure 2:**
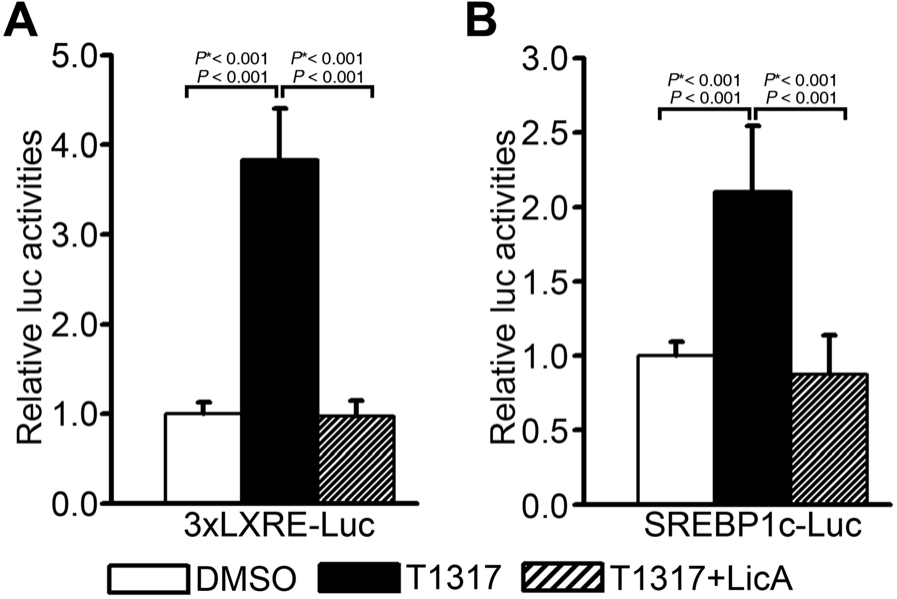
Effects of LicA on the T0901317-stimulated activation of LXRE-containing promoters. LXRα-mediated transcriptional activity was determined on **(A)** 3 × LXRE-luciferase or **(B)** SREBP-1c LXRE-luciferase reporters in the presence of 1 μM T0901317 and 10 μg/mL LicA in HepG2 cells. Luciferase activity was normalized to cotransfected β-galactosidase activity. Data were presented as means (SD) from four independent experiments with duplicate determinations. Statistical analysis was performed using one-way ANOVA. P* = *P* value for Bonferroni correction. DMSO, dimethyl sulfoxide; T1317, T0901317.

To confirm the inhibitory effects of LicA against LXRα activity in the context of the LXRE promoter without coercive DNA binding of Gal4-hLXRα LBD *via* the Gal4 DNA binding domain, we determined the T0901317-stimulated activity of LXRE-containing reporter genes in the presence of LicA. LicA inhibited not only T0901317-dependent LXR activation of the synthetic 3 × LXRE reporter (Figure 2A), but also of the natural SREBP-1c promoter (Figure 2B). These results indicated that LicA directly modulated transcriptional activation of the SREBP-1c gene *via* LXRE.

### LicA inhibits the LXRα-dependent expression of lipogenic genes and TG accumulation in primary hepatocytes

**Figure 3:**
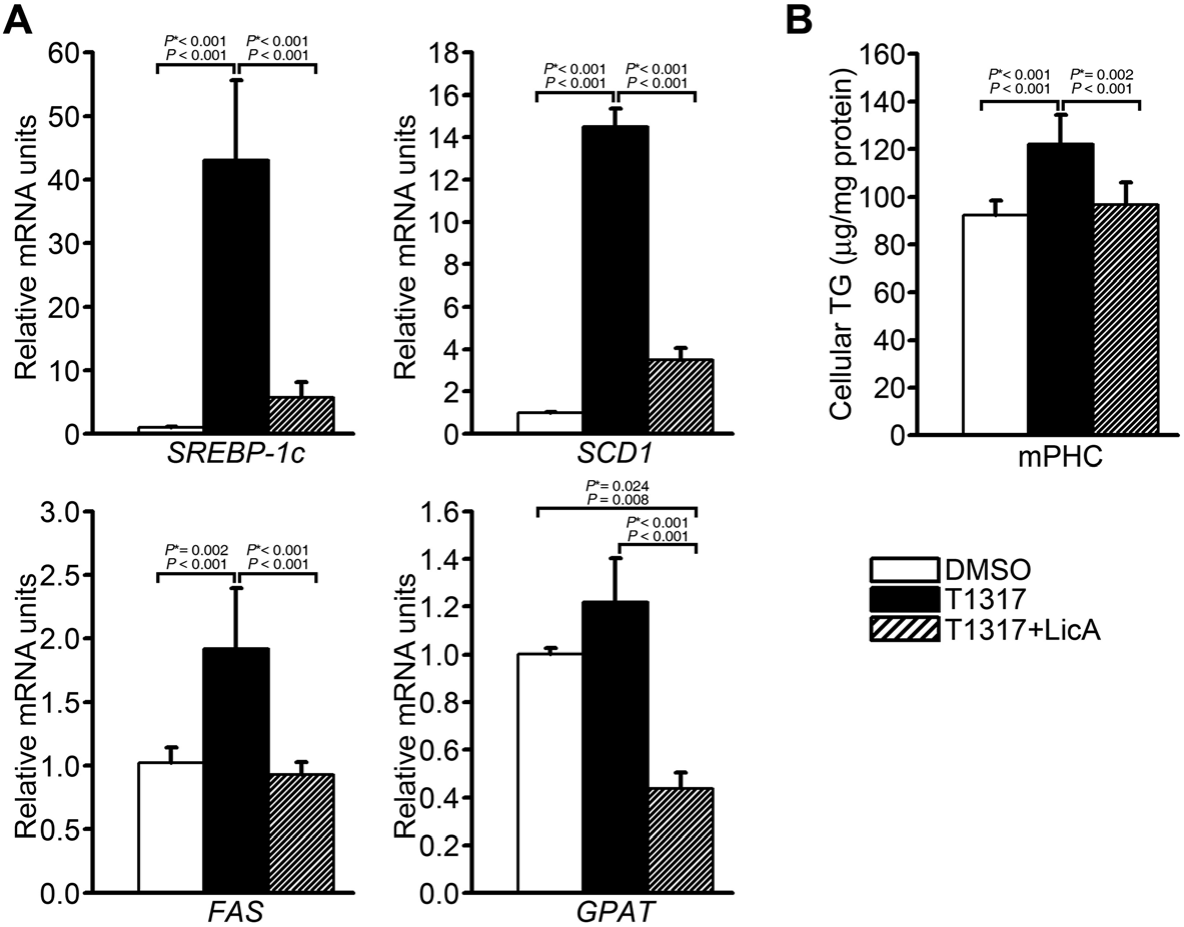
Effects of LicA on **(A)** the LXRα-stimulated expression of lipogenic genes and **(B)** TG accumulation in mouse primary hepatocytes. **(A)** T0901317-induced expression of SREBP-1c, SCD1, FAS, and GPAT was determined in the presence or absence of LicA by qRT-PCR. Data were presented as means (SD) from four independent experiments with triplicate determinations. **(B)** TG accumulation in response to cotreatment with T0901317 and LicA. Intracellular TG levels were measured enzymatically after treatment of primary hepatocytes with 1 μM T0901317 and 10 μg/mL LicA and normalized to total protein levels. Data were presented as means (SD) from four independent experiments with duplicate determinations. Statistical analysis was performed by one-way ANOVA. P* = *P* value for Bonferroni correction. DMSO, dimethyl sulfoxide; T1317, T0901317.

Because both LXRα and SREBP-1c activation enhance the transcription of many lipogenic genes, we examined the effects of LicA on the transcript levels of various lipogenic genes in primary hepatocytes by quantitative RT-PCR. LicA substantially repressed the T0901317-induced expression of SREBP-1c, FAS, SCD1, and GPAT (Figure 3A). SREBP-1c, FAS, and SCD1 are LXRα target genes, while FAS, SCD1, and GPAT are SREBP-1c target genes. Subsequently, cellular lipids were extracted and analyzed to determine whether LicA treatment altered the intracellular accumulation of TGs following LXRα activation. Quantitative analysis showed that LicA significantly reduced T0901317-stimulated TG accumulation in primary hepatocytes (Figure 3B). These results suggested that the inhibitory effects of LicA on LXRE-containing promoters decreased TG synthesis in primary hepatocytes.

### LicA does not inhibit the promoter activity and transcription of RCT-related LXRα target genes

**Figure 4:**
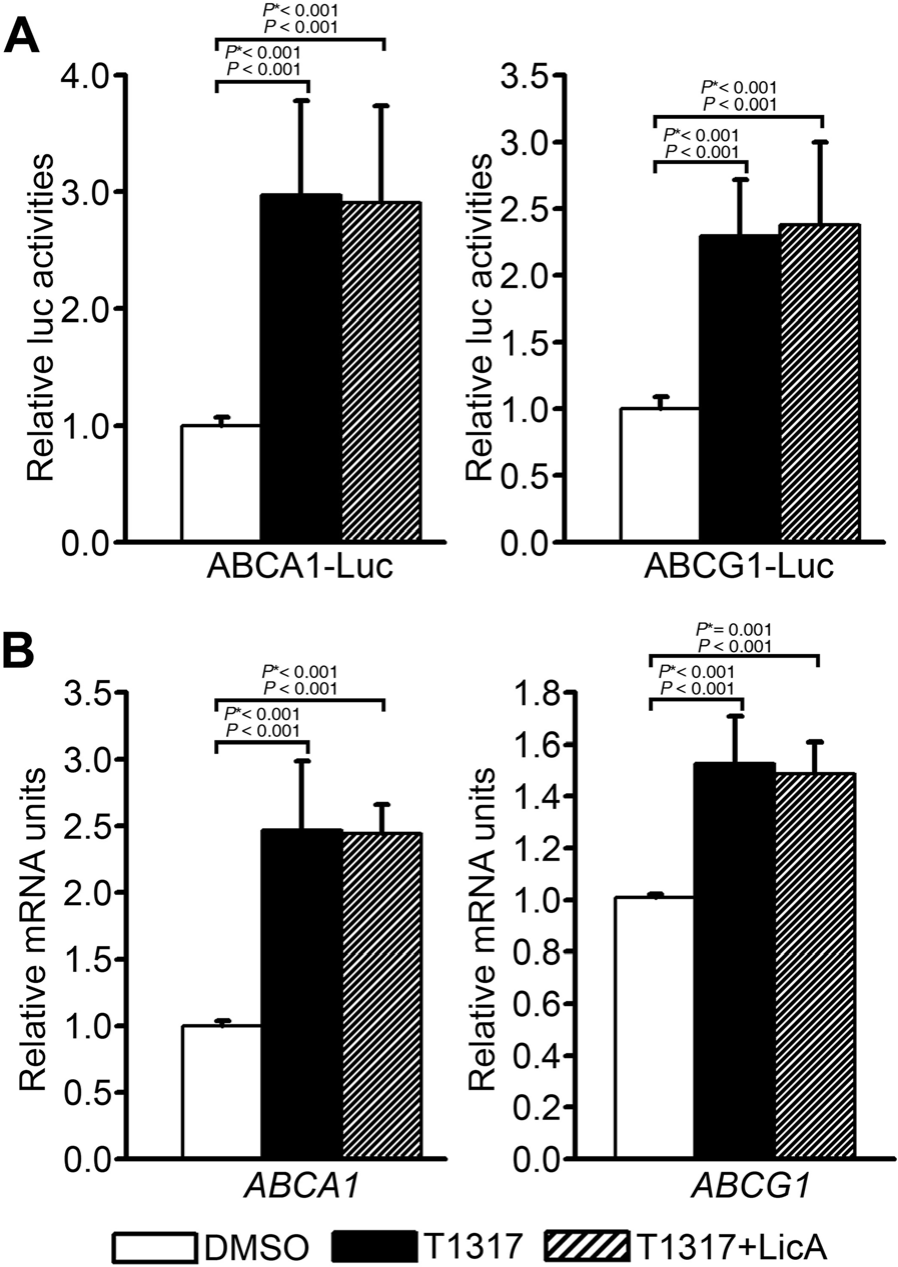
Effects of LicA on **(A)** the LXRE-containing promoter activity of ABCA1 and ABCG1 genes and **(B)** the T0901317-stimulated mRNA levels of ABCA1 and ABCG1. **(A)** The activity of ABCA1 LXRE-luciferase or ABCG1 LXRE-luciferase was determined, following treatment with 1 μM T0901317 and 10 μg/mL LicA, in HepG2 cells and normalized to cotransfected β-galactosidase activity. Data were presented as means (SD) from four independent experiments with duplicate determinations. **(B)** The mRNA levels of ABCA1 and ABCG1 were measured in primary hepatocytes treated with 1 μM T0901317 and 10 μg/mL LicA by qRT-PCR. Data were presented as means (SD) from 4 independent experiments with triplicate determinations. Statistical analysis was performed by one-way ANOVA. P* = *P* value for Bonferroni correction. DMSO, dimethyl sulfoxide; T1317, T0901317.

LXRα activation stimulates not only lipogenic genes, but also RCT-related genes such as ABCA1 and ABCG1 [23-25]. RCT is a process that transports cholesterol from peripheral tissues to the liver for excretion in the bile, improving metabolic diseases such as atherosclerosis. ABCA1 functions as the gatekeeper of the RCT process for eliminating excess cholesterol and has a major impact on cellular and whole body cholesterol metabolism [26]. ABCG1 also plays an important role in RCT *in vivo* by promoting cholesterol efflux onto lipidated apoA-I [24]. Therefore, we examined the effects of LicA on the promoter activity of ABCA1 and ABCG1 genes. LicA did not affect LXRα-dependent activation of ABCA1 and ABCG1 promoters (Figure 4A). To confirm this selective activity of LicA, we analyzed the effects of LicA on the transcript levels of ABCA1 and ABCG1 in the presence of T0901317 by quantitative RT-PCR. Consistent with the promoter assay, T0901317-dependent increases in ABCA1 and ABCG1 gene transcription were not repressed by LicA (Figure 4B).

### LicA selectively reduces the recruitment of RNA polymerase II to SREBP-1c LXRE, but not to ABCA1 LXRE

**Figure 5:**
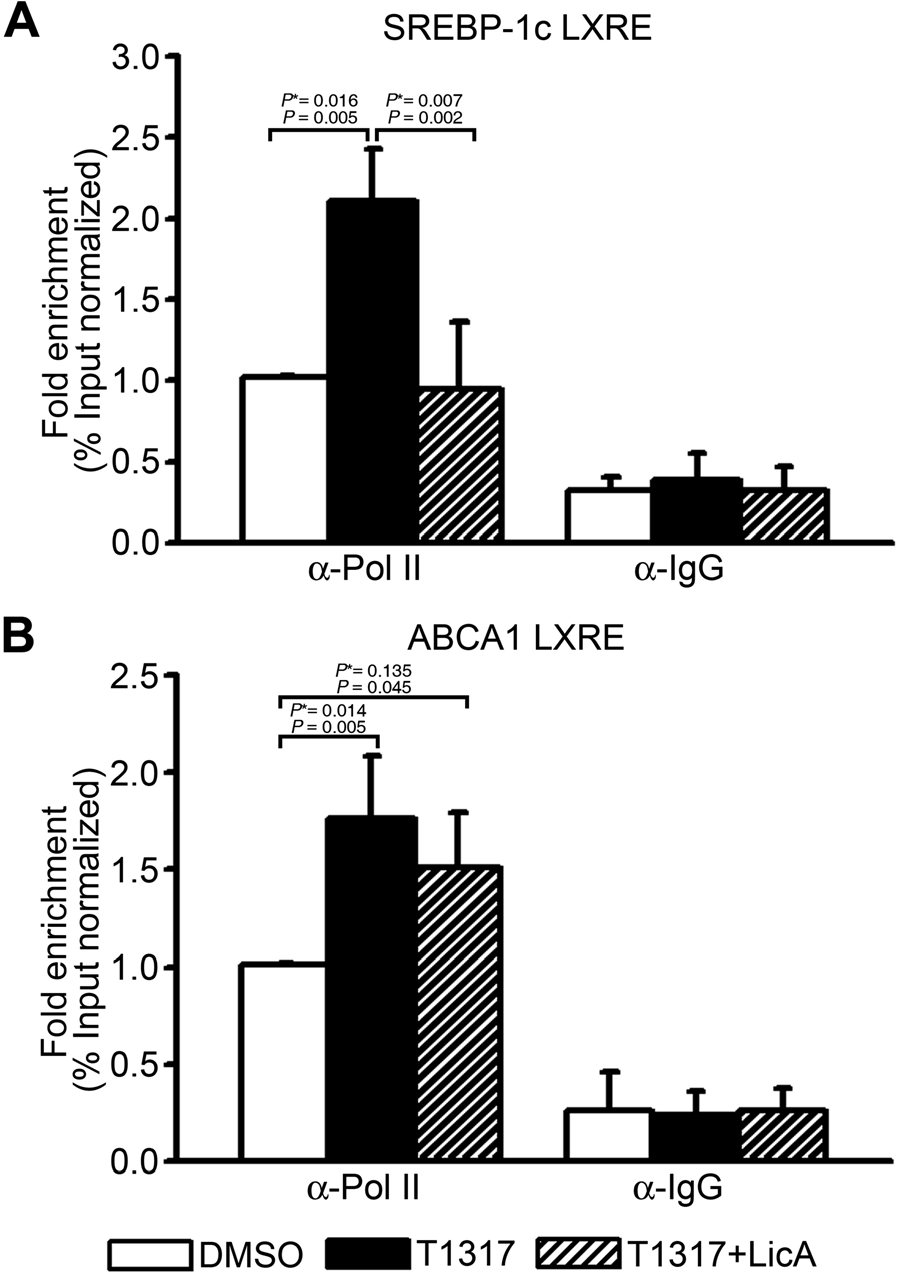
Effects of LicA on the recruitments of RNA polymerase II to the LXRE regions of SREBP-1c **(A)** and ABCA1 **(B)** genes. After treatment of primary hepatocytes with 1 μM T0901317 and 10 μg/mL LicA, ChIP assays were performed using anti-RNA polymerase II or IgG antibodies. Immunoprecipitated LXRE-containing DNA levels were determined by qRT-PCR and normalized to input DNA. Data were presented as means (SD) from four independent experiments with triplicate determinations. Statistical analysis was performed by one-way ANOVA. P* = *P* value for Bonferroni correction. DMSO, dimethyl sulfoxide; T1317, T0901317.

To elucidate the mechanism underlying the selective activity of LicA, we compared the recruitment of RNA polymerase II to the LXRE region of the SREBP-1c gene with that of the ABCA1 gene by a ChIP assay. In line with the previous results, the recruitment of RNA polymerase II to the LXRE region was inhibited on the SREBP-1c gene by LicA, but not on the ABCA1 gene (Figure 5). These results suggested that LicA inhibited SREBP-1c expression and lipid accumulation by selectively reducing the recruitment of RNA polymerase II to the LXRE region of the SREBP-1c gene.

## Discussion

LicA was identified to be an LXRα inhibitor in this study. LicA is one of the reversely constructed chalcones or “retrochalcones” (licochalcone A–E, echinatin) that are isolated and characterized from licorice, the root and stolon of the Glycyrrhiza plant (Leguminosae) (*e.g.*, *Glycyrrhiza glabra* or *Glycyrrhiza inflata*) [27-29]. It has been demonstrated that LicA has anti-inflammatory [30,31], antiparasitic [32], antifungal [33], anticancer [34,35], and osteogenic activities [36]. Recently, Quan *et al.* [37] reported that LicA inhibited SREBP-1c and its target genes *via* indirect activation of AMP-activated protein kinase and showed antilipogenic effects in the liver. Based on our current results, we suggested that LicA also directly inhibited SREBP-1c transcription in hepatocytes.

Transcription of SREBP-1c is activated by insulin in the liver through the combinatorial actions of SREBP, LXR, specificity protein 1, and nuclear factor Y [38]. Among these factors, LXR plays a central role in the insulin-mediated activation of SREBP-1c and subsequent stimulation of fatty acid synthesis [17]. In the present study, LicA was initially selected for its inhibitory effect against autonomous transactivity of LXRα. Further investigation showed that LicA acted directly on the LXRE of the SREBP-1c gene. LicA inhibited the recruitment of RNA polymerase II to the LXRE region of the SREBP-1c gene, thus repressing LXRα-dependent expressions of SREBP-1c and its target genes related to lipogenesis. All these actions together resulted in diminished accumulation of TG in primary hepatocytes following treatment with T0901317.

**Figure 6:**
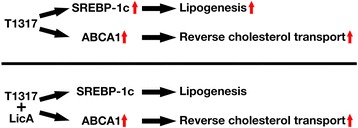
Schematic diagram of selective inhibition of lipogenic pathway by LicA. Combination therapy of LicA and T0901317 could improve atherosclerosis without lipogenic adverse effects.

The inhibition of LXRα-mediated transcription by LicA was selective for lipogenic process (Figure 6). It is well known that LXRα regulates both lipogenic genes and RCT-related genes by direct binding to their LXREs [18,39,40]. Nevertheless, LicA selectively inhibited the transcription of the lipogenic SREBP-1c gene, but did not repress the LXRα-stimulated transcription of RCT-related genes including ABCA1 and ABCG1. The recruitments of RNA polymerase II to the LXREs were differentially regulated by LicA between SREBP-1c and ABCA1 promoters.

ABCA1 and ABCG1 promote cellular cholesterol efflux synergistically. The efflux of cholesterol and phospholipids to apoA-I mediated by ABCA1 converts apoA-I into nascent high density lipoprotein, which can then act as an efficient acceptor for ABCG1-mediated cholesterol efflux, followed by formation of mature HDL particles [41-43]. Hence, the selective inhibitory activity of LicA against the lipogenic pathway could be therapeutically beneficial for the treatment of metabolic diseases. Further investigation is required to identify the factor by which the recruitments of RNA polymerase II to LXREs are different between SREBP-1c and ABCA1 promoters. It is a noteworthy fact that SREBP-1c and ABCA1 are differently regulated in LXR^-/-^ macrophages through the promoter-specific roles of LXR/corepressor complexes [44].

## Conclusion

LicA is a selective inhibitor of LXRα, repressing lipogenic LXRα target genes but not RCT-related LXRα target genes.

## Abbreviations

ABCA1: ATP-binding cassette transporter A1
ABCG1: ATP-binding cassette transporter G1
ACC: Acetyl-CoA carboxylase
ChIP: Chromatin immunoprecipitation
FAS: Fatty acid synthase
GPAT: Glycerol-3-phosphate acyltransferase
LicA: Licochalcone A
LXRα: Liver X receptor α
LXRE: LXR response element
RCT: Reverse cholesterol transport
SCD1: Stearoyl-CoA desaturase 1
SREBP-1c: Sterol regulatory element binding protein-1c
TG: Triglyceride
